# LUMINEX®: a new technology for the simultaneous identification of five *Entamoeba* spp. commonly found in human stools

**DOI:** 10.1186/1756-3305-6-69

**Published:** 2013-03-15

**Authors:** Helena Lúcia Carneiro Santos, Kakali Bandyopadhyay, Rebecca Bandea, Regina Helena Saramago Peralta, José Mauro Peralta, Alexandre Januário Da Silva

**Affiliations:** 1 Instituto de Microbiologia da Universidade Federal do Rio de Janeiro, Rio de Janeiro, Brazil; 2 LAPSA, Instituto Oswaldo Cruz, Fundação Oswaldo Cruz, Rio de Janeiro, Brazil; 3 Center for Global Health, Division of Parasitic Diseases and Malaria, Centers for Disease Control and Prevention, Atlanta, GA, USA; 4 Departamento de Patologia, Universidade Federal Fluminense, Niterói, RJ, Brazil

**Keywords:** Entamoeba, 18SrRNA, PCR, Molecular diagnostics, Multiplex assay

## Abstract

**Background:**

Six species of the genus *Entamoeba*, i.e., *E. histolytica, E. dispar, E. moshkovskii, E. polecki, E. coli, and E. hartmanii* can be found in human stools. Among these, only *E. histolytica* is considered to be pathogenic, causing intestinal and extra-intestinal disease, but it is morphologically identical to *E. dispar* and *E. moshkovskii*. In general, *E. polecki*, *E. coli*, and *E. hartmanii* can be differentiated morphologically from *E. histolytica*, but some of their diagnostic morphologic features may overlap creating issues for the differential diagnosis. Moreover, the previous inability to differentiate among *Entamoeba* species has limited epidemiologic information on *E histolytica*. The objective of this study was to develop a rapid, high-throughput screening method using Luminex technique for the simultaneous detection and differentiation of *Entamoeba* species.

**Methods:**

PCR amplification was performed with biotinylated *Entamoeba* sp 18S rRNA gene primers, designed to amplify a fragment ranging from 382 to 429 bp of the *Entamoeba* spp studied. Regions of this fragment that could differentiate among *E. histolytica*, *E. moshkovskii, E. dispar, E. hartmanii* and *E. coli* were selected to design hybridization probes to link to Luminex beads. The assay was standardized with cloned DNA samples of each species and evaluated with 24 DNA extracts from samples obtained from individuals diagnosed with these amebas in their stools.

**Results:**

Using this approach we were able to correctly identify *E. histoltyica*, *E. dispar*, *E hartmanni*, *E. coli* and *E. moshkovskii* in all specimens studied. From twenty four samples tested by microscopy, PCR/DNA Sequencing and real-time PCR, 100% agreed with PCR-Luminex assay for identification of *E. dispar, E. moshkovskii, E. hartmanni, E. histolytica,* and *E. coli.*

**Conclusion:**

These results show that this method could be used in the diagnostic detection of *Entamoeba* spp in fecal samples. This diagnostic test was useful to clearly distinguish E histolytica from other species and also to strengthen epidemiologic data on *Entamoeba* spp.

## Background

The genus *Entamoeba* contains many species, some of which (ie, *E histolytica, E. dispar, E. moshkovskii, E. polecki, E. coli* and *E. hartmanni*) can be found in human stools [1,2]. *E. histolytica*, thus far, is the only species associated with disease [2,3]. *E. histolytica* may cause invasive disease and extra intestinal amebiasis. It is also evident that not all humans infected with *E. histolytica* develop clinical disease and in most cases, it may cause mild or asymptomatic infections [4].

Amoebas may cause a variety of clinical presentations, from asymptomatic commensal colonization to invasive amebic dysentery and extraintestinal infections. Infected individuals may be initially asymptomatic and develop symptoms later in the course of the infection. Previous studies have estimated that only one in four *E. histolytica* infections progresses to symptomatic [5-8]. Therefore, WHO recommends that *E. histolytica/E. dispar* should be differentiated whenever it is possible and such patients should not be treated on the basis of microscopy findings alone. Yet, regardless of symptoms, all cases presumptively diagnosed or confirmed as being caused by *E. histolytica*, should be treated to minimize the risk for progression to invasive disease. On the other hand, cases confirmed to involve only *E. dispar* should not be treated. If a patient with *E. dispar* or *E. moshkowskii* has intestinal symptoms, a further investigative search should be made to diagnose other potential causes and in some cases treatment with drugs effective against protozoan parasites will be implemented; e.g. when no other causes are identified. This is because the traditional methods used to diagnose amebic infections available in clinical laboratories may fail in providing a correct identification to the species level of ameba parasites. Asymptomatic *E. histolytica* infection should be treated with a luminal amoebicide (diloxanide furoate or paromomycin), and invasive intestinal or extra-intestinal amebiasis should be handled by administering a tissue amoebicide (metronidazole) followed by luminal treatment (WHO, 1997).

The other *Entamoeba* species are considered as commensal or non-invasive forms, where no symptoms are present. Nevertheless, all *Entamoeba* spp. found in human stools should be reported in the parasitological examination. *E. hartmanii* can be differentiated from other morphologically similar species primarily based on size. *E. poleckii* and *E. coli* can be differentiated morphologically from others*,* but some of their diagnostic morphologic features may overlap, depending on the condition of the specimen [9]. The diagnosis of amebiasis and the identification of *Entamoeba* spp. to the species level are routinely performed by the identification of the parasite’s morphologic features ascertained through the examination of stained stool smears. This task might be challenging, considering that precise identification of such diagnostic morphologic features require advanced expertise. Despite all the issues stressed above, the differentiation of *E. histolytica, E. dispar* and *E. moshkovskii* in the stool samples is the main limitation of microscopy-based diagnosis. Cyst and trophozoite stages of these species are morphologically identical. However, all *Entamoeba* species can be differentiated at the molecular level [10]. Laboratory tests that have been developed to diagnose amebiasis have focused on the detection of parasite antigen in the feces by the use of monoclonal antibodies or based on the detection of parasite DNA by PCR amplification. A few commercial ELISA kits are available for detection of *E. histolytica,* such as the TechLab *Entamoeba* test to detect *E. histolytica/E. dispar *[11], Alexon ProSpecT ELISA to detect *E. histolytica/E. dispar* and *Giardia lamblia*[12] and a Triage parasite panel to detect antigen of *E. histolytica/E. dispar*, *Giardia lamblia* and *Cryptosporidium parvum* in stool specimens [13]. The main limitation of all these ELISA kits is that they can identify the amoebae only as *E. histolytica*/*E. dispar* complex but not specifically as *E. histolytica*, *E. dispar* or *E. moshkovskii*. However, a monoclonal antibody based Tech Lab *E. histolytica* II ELISA is commercially available for the specific detection of *E. histolytica* antigen directly in stool specimens [11]. During the last decade, a remarkable development in molecular biology-based procedures to detect *E. histolytica* took place. A wide variety of PCR methods targeting different genes, including 18S rRNA gene, genes that codify for the 30-kDa antigen, serine-rich protein, chitinase, hemolysin, and the extra-chromosomal circular DNA, have been described for the detection and differentiation of *E histolytica, E dispar *[14-21] and more recently *E. moshkovskii* in human stools [22-24]. However, some of these studies reported false negative results when these techniques were compared to microscopy examination, most of the time when other *Entamoeba* species; e.g., *E. hartmanii*, *E. poleckii*, *E. coli*, were present [7,25-28]. DNA based approaches can be multiplexed to allow identification of multiple organisms simultaneously. Recently, there have been an increasing number of multiplex assays in the literature, such as PCR followed by multi-analytical hybridization using fluorescent microspheres as solid supports coupled with flow cytometry. This technique is able to detect multiple DNA targets in a single reaction tube. This is possible by the use of sets of microspheres coupled to probes that hybridize to complementary PCR-amplified DNA targets. In the Luminex platform, the hybridized strands on these microspheres are fluorescently tagged and the beads are individually analyzed with a red laser that recognizes the microsphere set, and a green laser that provides readout of the bound DNA target. This method has been used for the detection and differentiation of several species of bacteria, fungi, virus and protozoa [20,29-39]. A Luminex assay for detection of intestinal parasite DNA was recently standardized, including *E. histolytica*[40]. This assay afforded between 83 to 100% of sensitivity and specificity in comparison to real-time PCR.

In this study we describe the development of a multiplex direct hybridization assay using a Luminex technology, for rapid simultaneous detection of *E. histolytica, E. dispar, E. hartmanii, E. moshkovskii and E.* coli. Rather than conduct a formal evaluation of the molecular approach, we focused on demonstrating proof of concept. This approach can be used as a diagnostic method to strengthen epidemiologic data by making it more feasible to identify mixed infections.

## Methods

### Control samples

Plasmid DNA containing 18S-rRNA sequences of *E. histolytica, E. dispar, E. moshkovskii, E. hartmanii* and *E. coli* were used for the initial standardization of the PCR-based suspension array assay. Amplified PCR products generated with primers JVF/DSPR2 were purified with StrataPrep PCR Purification Kit (Stratagene, La Jolla, CA) and cloned using pCR2.1-TOPO vector as described in the protocol from the TOPO TA cloning Kit (Invitrogen, Carlsbad, CA,USA) [10].

### Samples

A total of 74 DNA extracts from human stools were used to evaluate the approach. Nine of these DNA extracts were from stools that were positive for *Entamoeba* spp by microscopy, obtained from Brazilian patients. Fifteen additional DNA samples extracted from positive stools sent to CDC for confirmatory diagnosis of amoebiasis were used in this study and fifty stool samples with negative direct parasitological examination were included as a control. All parasitological positive samples were tested using real-time PCR and nine Brazilian stools samples were also analyzed by DNA sequencing as described elsewhere [10,20]*.* In addition, the specificity of PCR-LUMINEX assay was evaluated by using 11 DNA samples from other intestinal parasites: *Endolimax nana* (n=1), *Blastocystis hominis* (n=4), *Giardia intestinalis* (n=2), *Microsporidia* (n=1), *Cryptosporidium parvum* (n=2) and *C. hominis* (n=1).

### DNA extraction

Total genomic DNA from the clinical samples was extracted from 300 to 500 μl of human fecal samples, using the FastDNA method (MP Biomedicals, Solon, OH) combined with the QIAquick PCR purification kit (QIAGEN Inc., Valencia, CA) as described previously, [41]. Purified DNA was stored at 4°C until used for the molecular analysis.

### PCR amplification

To amplify the fragments from the 18S rRNA gene, we used the sets of primer, JVF/DSPRS2, JVF/EntaREV 390 and JFV/Enta417 (Table 1). The reverse primers were synthesized with biotin at the 5′ extremity to allow detection of hybridized amplicons with fluorescent streptoavidin moieties. PCR reactions were performed in a 50 μl-volume containing 20 mM of Tris–HCl pH 8.4; 50 mM of KCl; 1.5 mM of MgCl_2_; 12 pmoles of each oligonucleotide primer JVF/EntaREV 390; 250 μM of each deoxynucleoside triphosphate (dNTPs) and 1.25 U of Taq DNA polymerase (Invitrogen) and 10 μl of DNA sample undiluted and diluted at 1/10. The PCR amplification reactions were carried out in a Veriti 96 well thermal cycler (AB Applied biosystems, Foster City, CA, USA); assay details such as cycling structure and sequence of primers are outlined in Table 1. Amplified products were resolved by electrophoresis in a 2.0% of agarose gel containing 0.5 μg of ethidium bromide/ml.

**Table 1:** PCR primers and conditions

**Generic PCR**	**Primers (5′ -3′)**	**Cycling structure**
JVF (forward)	GTTGATCCTGCCAGTATTATATG	95°C for 5 min followed by 40 cycles of 95°C for 30 s, 57°C for 30 s, 72 C for 1 min, 72 C for 7 min
DSPR2 B (reverse)	CACTATTGGAGCTGGAATTAC	
JVF (forward)	GTTGATCCTGCCAGTATTATATG	95°C for 5 min followed by 40 cycles of 95°C for 30 s, 50°C for 30 s, 72 C for 1 min, 72 C for 7 min
EntaRev 390* (reverse)	ATTCCTCGTTATCCGTTAT	
JVF (forward)	GTTGATCCTGCCAGTATTATATG	95°C for 5 min followed by 40 cycles of 95°C for 30 s, 55°C for 30 s, 72 C for 1 min, 72 C for 7 min
EntaRev417* (reverse)	AAAGCTCCTCTCCGATGT	

*tagged with biotin at the 3′.

### Probe design

DNA sequences of *E. histolytica*, *E. dispar*, *E. moshkovskii*, *E. hartmanni* and *E. coli* deposited in The Genetic sequence database at the National Center for Biotechnical Information (NCBI) (GenBank), under accession numbers X64142, AB197936, Z49256, AF149906, AF149907, AF149915 and AF149915, respectively, were used to design the specific hybridization probes. These sequences were aligned in the GeneStudio suite (GeneStudio, Inc. Suwannee, GA). Probes were pre-selected based on results of Basic Local Alignment Search Tool (BLAST) searches used to verify potential cross-hybridization with other microorganisms (National Center for Biotechnology Information, Bethesda, MD; http://www.ncbi.nlm. nih.gov) [42]. Oligonucleotide probes were synthesized with an amino-modified group at the 5′ end and linked to a 6-carbon linker, as described elsewhere [43]. Each probe was covalently linked to a specific Luminex microsphere classification. Signals were only generated when biotinylated sequences bound to the complementary probe on the respective microsphere classification. Secondary structure of the probes was verified by using the DNA folding application (http://mfold.bioinfo.rpi.edu/cgi-bin/dna-form1.cgi). To design the probes, regions prone to secondary structure were avoided when possible. Probes that would not give the expected hybridization signal were discarded and new probes were synthesized and re-tested. The list of probes designed for specific detection of PCR products are shown in Table 2.

**Table 2:** List of probes used in this study

**Specificiy(ies)**	**Probes**	**Probe sequence (5′ – 3′)**	**Length (nt)**
*E. histolytica*	Hist 1	TAGTACAAAATGGCCAATT	19
*E.histolytica*	Hist 116	GGTTAGTAAAATACAAGG	18
*E. histolytica*	Hist 168	CGATCCAGTTTGTATTAGT	19
*E. histolytica*	Hist 200	TATTAGTACAAAATGGCCAAT	21
*E. histolytica*	Hist 242	AATGAATTGAGAAATGACAT	20
*E. dispar*	Disp1	ACGATCCAATTTGTATT	17
*E.dispar*	Disp 2	GTTAGAGATTAAGCCAT	17
*E.dispar*	Disp 3	TAGAGATTAAGCCATGC	17
*E.dispar*	Disp 4	ATGTTAGAGATTAAGCCA	18
*E. dispar*	Disp186	GACGATCCAATTTGTATT	18
*E. dispar*	Disp 238	GTAAGTAAATTGAGAAATGAC	21
*E. moshkovskii*	Emosh 1	AGACGATCCGGTTTGTAT	18
*E. moshkovskii*	Emosh 2	TAAATACTCTTACGAAATC	19
*E. moshkovskii*	M1	GTATGACAATTGTAGAGC	18
*E. moshkovskii*	M2	ATGGTATGACAATTGTAGA	19
*E. moshkovskii*	M3	GACAATGTAGAGCACACAG	19
*E. hartmanni*	Ehart 1	ATGAGAATATCTGATCTA	18
*E. hartmanni*	Ehart 123	ATTAGTAAGTACAAGGAT	18
*E. coli*	Ecoli 165	TGACGGTTTTCACCCCTT	18
*E. coli*	Ecoli 310	AGAGATTTTCACAAGTCA	18
*E.histolytica and E. dispar*	Hist/Disp 275	TTAGGATGCCACGACAATT	19
*E. hist, E. disp, E. coli, E. hart*, *E. mosh*	EGP 1	TACAGGATAGCTTTGTGAAT	20
*E. hist, E. disp, E. coli, E. hart*, *E. mosh*	EGP 2	TGAATGATAAAGATAATACT	20

### Probe coupling

Briefly, the capture probes modified at the 5′ end with 5-carbon linker and amine were covalently coupled to carboxylated microspheres (Luminex Corp, Austin, TX, USA) using a carbodiimide coupling procedure. Individual sets of microspheres were prepared by placing 200 μl of stock suspension in a 1.5 ml microcentrifuge tube, the microspheres were suspended by sonication and vortexed for approximately 30 seconds, followed by a centrifugation at 10,000 x g for 1 minute. The supernatant was discarded and the beads were suspended in 50 μl of 0.1 M MES (2-N-morpholino-ethanesulfonic acid, Sigma, St Louis, MO, USA), pH 4.5, using 5 N NaOH. Once suspended, the beads were vortexed and 2 nmoles of the distinct oligonucleotides were added to the bead mixtures. 2.5 μl of 30 mg/ml freshly prepared *N*-(3-Dimethylaminopropyl)-*N’*-ethylcarbonate (EDC) was immediately added to each bead-probe mixture to allow the attachment of the amine modified probe to the carboxylated beads. The microsphere mixtures were incubated in the dark for 30 minutes with continuous shaking. Incubation was repeated using a fresh 30 mg/ml solution of EDC and the microspheres were washed once with 1 ml of 0.02% polyxyethylenesorbitan monolaurate (Tween 20), the beads were vortexed and then centrifuged at 10,000x g for 1 minute. The supernatant was removed and 1 ml of 0.1% sodium dodecyl sulfate (SDS) was added and the mixture was vortexed once more. Mixtures were centrifuged at 10,000x g for 1 minute and the supernatant was once again discarded. Coupled microspheres were stored in 50 μl TE buffer (10 mM Tris- HCl, 1 mM EDTA, pH 8.0) in the dark at 4°C.

### Hybridization assay procedure

The hybridization assay was based on the binding of the complementary 5′ biotin labeled PCR amplicons to specific capture probes designed on DNA sequences that could discriminate among *E. coli*, *E. histoltyica, E dispar, E, hartmanii,* and *E, moshkovskii*. In addition, an *Entamoeba* genus specific probe was used (Table 2). The assay was performed in a 96-conical well plate (Costar, Corning, NY, USA). The total reaction volume was 50 μl, which included 33 μl of microsphere mixture and 17 μl of the amplified product or TE buffer, which was used a blank control. To prepare the microsphere mixture, the volume of each microsphere set was calculated and added to 1.5× TMAC buffer (4.5 M tetramethylammonium chloride, 75 mM Tris–HCl, pH 8.0, 6 mM EDTA, and 0.15% sarkosyl) to achieve a concentration of 1500–2500 microspheres per set in a final volume of 33 μl. PCR products were added to wells, the titer plate was sealed and the amplified DNA was denatured at 95°C for 5 min, followed by incubation at 41°C, 43°C, 46°C, 48°C, 50°C, and 52°C for 45 to 60 minutes in a Veriti 96 well thermal cycler (AB Applied biosystems). After this incubation, 25 μl of a 1:50 and 1:80 dilution of pre heated R-phycoerythrin conjugated strepavidin 1 mg/ml (SA-PE Molecular Probes, Eugene, OR, USA) was added to each well. The plates were tapped and the content of the wells pipetted up and down. The samples were then incubated for an additional 5, 10 and 15 min at the same hybridization temperature and then analyzed using the Luminex platform. Data acquisition, Xponent software v. 3.0 (Luminex Corp) was used for the analysis. Each sample was run in duplicate with four blank controls per plate. The median fluorescent intensity (MFI) of the SA-PE conjugate bound to 100 of each microsphere population was reported. The MFI values for samples were corrected by subtracting the average values of the blank controls. Cloned samples were used to evaluate the reproducibility of the assay in five independent repeats.

### Ethical approval

This study was reviewed and approved by the Human Investigation Committee of Universidade Federal Fluminense, Niteroi, Brazil with protocol nº 020/07 for Brazilian samples. All CDC DNA samples used in this study were anonymized after submitted to CDC for confirmatory diagnosis from state public health laboratories, hospitals and private clinics in the United States.

## Results

**Figure 1:**
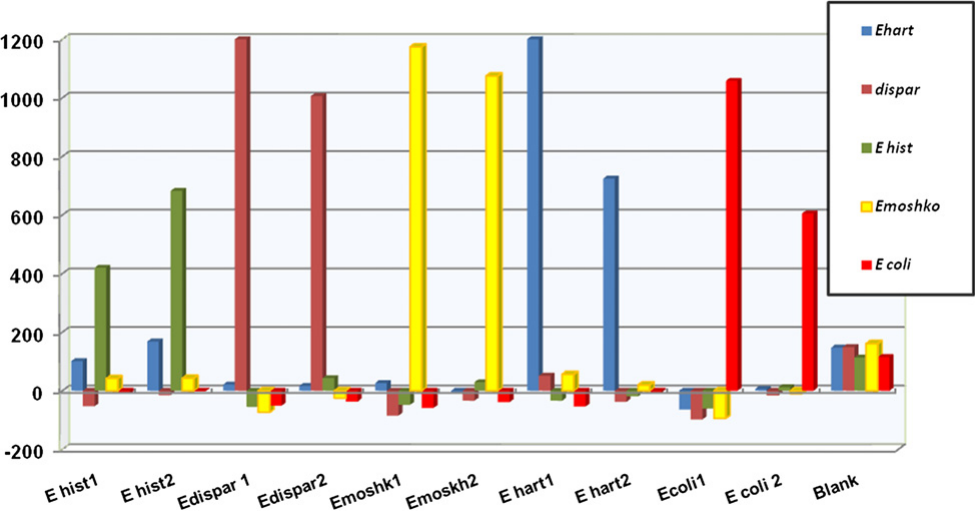
**Represents hybridization signals obtained from cloned-derived PCR biotinylated products from five *Entamoeba *species.** (1) 5 μl of biotinylated amplicon diluted with 12 μl of TE buffer (pH 8) and (2) 17 μl of biotinylated amplicon diluted 1:100 in TE were used to determine the optimal conditions for hybridization and demonstrate that maximal MFI values were obtained with a hybridization temperature of 46°C, hybridization time of 60 min, SA-PE at a dilution of 1:50 preheated. Strains and probes tested were as follows: Microsphere coated with hist116 *(E. histolytica),* Dis 238 (*E. dispar*), Ecoli310 (*E. coli*), Ehart1 (*E. hartmanni* ) and Emoshk (*E.moshovskii*).

**Figure 2:**
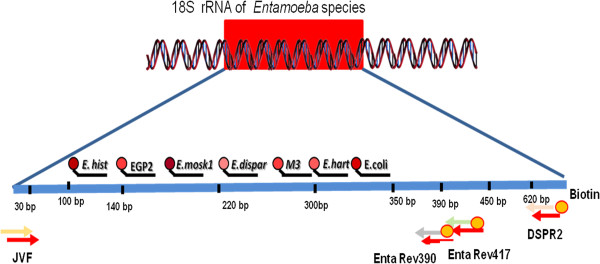
**Location of the probes in strand DNA target.** Hist 116, Disp 238, M3 and Emoshk1, Ecoli310, Ehart1 and EGP2 linked on specific microsphere set hybridized in the region complementary of their respective biotinylated strand DNA target.

The preliminary experiments evaluated the effectiveness of the primers JVF/DSPR2 targeted to amplify a conserved region of 18 S rRNA of *Entamoeba* species, which produced amplified products ranging from 622 to 667 bp, depending on the species. In this step cloned DNA samples were amplified from 86 ng/ml *E. moshkovskii*, 28 ng/ml of *E. dispar*, 46 ng/mL of *E. histolytica*, 26 ng/ml of *E*. *hartmanni* and 54 ng/mL of *E. coli*. No amplification was produced when DNA extracted from *Endolimax nana* (n=1), *Blastocystis hominis* (n=4), *Giardia intestinalis* (n=2), *Microsporidia* (n=1), *Cryptosporidium parvum* (n=2) and *C. hominis* (n=1) were used. To test the multiplex capability of the Luminex technology®, beads with probes coupled to them were pooled together and tested using a single target per well. The biotinylated PCR amplicons were then hybridized with a panel of probes linked to specific microsphere classifications to *E. dispar* (n=6), *E. histolytica* (n=5), *E. hartmanni* (n=2), *E. moshkovskii* (n=5) and *E. coli* (n=2) as well as group specific probes (n= 3) (Table 1). Preliminary experiments were designed to evaluate the specificity of the probes and the stringency conditions necessary to discriminate among the different species. The optimal conditions of hybridization assays were determined after a systematic comparison of different hybridization temperatures, incubation times, SA-PE concentrations. Hybridization experiments were performed testing different incubation temperatures; i.e., 40°C, 45 °C; 50°C and 55°C for 30, 45 or 60 minutes. The SA-PE was tested under dilutions of 1:20, 1:80 and 1:50 in 1.5 × TMAC buffer using incubations of 10 and 15 min. The results of hybridization tests showed low median fluorescence intensity (MIF) for all probes, in which intensity of signals were close to background value. We observed that this was due to the presence of the secondary structure on the biotinylated strand DNA target located in the region complementary to the sequences of the probes. Novel reverse primers were designed targeting the same region. The hybridization assays with the plasmid-derived PCR biotinylated products produced by new primers JVF/EntaRev390 and JVF/Enta417B showed optimum hybridization signal at 46°C for 60 minutes (Figure 1). Followed by the addition of 25 μl of streptavidin-R-phycoerythtrin solution pre heated in a dilution of 1:50 in 1 X TMAC buffer, incubated at 46°C for 5 minutes in the dark and analyzed on the Luminex platform ®. SA-PE at a 1:50 in 1X TMAC buffer produced maximum signals with minimum background (Figure 1). These conditions provided optimal discrimination between perfect matched and mismatched sequences, generating highest MIFs for *E. dispar, E. moshkovskii, E. hartmanni, E. coli*, except for *E. histolytica,* which displayed median MIF (Figure 1) when compared to background. From 23 probes analyzed only 7 showed a good fluorescence profile, the probes Hist 116, Dis 238, M3 and Emoshk1, Ecoli310, Ehart1 and EGP2 hybridized with their respective targets (Figure 2), with no cross-hybridization. Non-specific hybridization was seen when the assay was performed at 41°C and 43°C for 60 minutes. Increasing the hybridization temperature to 48°C reduced the discrimination profile of *E. dispar, E. moshkovskii,* and *E. histolytica* when specific probes were used. Two different dilution ranges of the samples were evaluated in this study. Amplicons were tested using 5 μl of biotinylated amplicons diluted with 12 μl of TE buffer (pH 8) and 17 μl of biotinylated amplicon diluted 1:100 in TE. Four blank samples were used in every run. A background well, consisting of all reaction components except a DNA template was used to determine the background level of the reporter fluorescence associated with each bead. MFI final results were reported by subtracting the background MFI values from the sample MFI values. Once the multiplex PCR based suspension array parameters were determined using the cloned samples of *Entamoeba*, this approach was evaluated with twenty-four DNA extractions obtained from positive clinical specimens. PCR amplification was performed with biotinylated *Entamoeba* sp 18S rRNA gene primers JVS/ Rev417B, designed to amplify a fragment ranging from 382 to 429 bp. From these, nine samples were tested by microscopy, PCR/DNA Sequencing and real-time PCR tests. The results obtained from these samples demonstrated an agreement of 100% among Luminex and PCR/DNA sequencing for identification of *E. dispar, E. moshkovskii, E. hartmanni, E. histolytica,* and *E. coli* (Table 3), as well as real-time PCR specific for *E. histolytica* and *E. dispar*. We were able to identify samples with more than one species of *Entamoeba* by performing a Luminex assay. All fifty parasitologically negative stool samples were negative in the assay. Of the additional 15 DNA extractions from stool samples tested, five samples were positive for *E. histolytica*, five were positive for *E. dispar* and five were negative by real time PCR. The results of the Luminex assay from these samples showed 100% of concordance with the real-time PCR. These data demonstrated that the assay was 100% specific for *E. histolytica* and *E. dispar.* Some of the samples were positive for *E. hartmanii* by Luminex assay. The results are shown in Table 4.

**Table 3:** Results from real time PCR and DNA sequencing compared with PCR based suspension array for nine stool samples

**Stool samples**	**Real time PCR**	**DNA sequencing**	**PCR based suspension array assay**
02/Br	*E.dispar*	*E.dispar*	*E.dispar; E.coli*
03/Br	*E.dispar*	*E.dispar*	*E.dispar*
05/Br	*E.dispar*	*E.dispar*	*E.dispar*
06/Br	*E.dispar*	*E.dispar*	*E.dispar*
15 br	*Negative*	*E.coli*	*E. coli*
17/Br	*E.dispar*	*E.dispar; E.hart*	*E.dispar; E.hart*
18/Br	*Negative*	*E.hatmanni*	*E.hartmanni*
19/Br	*E.dispar*	*E.dispar; E.hart*	*E.dispar; E.hart*
28/Br	*Negative*	*E.coli*	*E. coli; E.hart*

**Table 4:** Results of Luminex assay and real time PCR with 15 DNA from stool samples

	***E.dispar***	***E. hist***	***E. moshk***	***E hart***	***E.coli***	Real time PCR^**^
CTL+ Eh_ 1^*^	−41	981	93	140	−89	-
CTL+ Ed_1	1368	54	−61	214	−79	-
CTL+ Em_ 2	−76	−48	547	−45	−86	-
CTL+ Ehart_1	−82	−87	−80	1844	−85	-
CTL+ Ecoli	−75	−60	−84	−53	1194	-
Sample 1	530	−15	−64	181	−73	*E.dispar*
Sample 2	772	−10	−74	728	−76	*E.dispar*
Sample 3	−77	−76	−71	−79	−68	Negative
Sample 4	−44	1094	135	201	59	*E. hist*
Sample 5	−43	1144	154	185	75	*E. hist*
Sample 6	597	−4	−75	578	−80	*E.dispar*
Sample 7	−79	−70	−77	−89	−87	Negative
Sample 8	−50	1027	101	172	44	*E. hist*
Sample 9	−39	1144	117	197	49	*E. hist*
Sample10	−39	1145	172	255	97	*E. hist*
Sample 11	−85	−69	−86	−94	−93	Negative
Sample 12	−83	−84	−91	−95	−88	Negative
Sample 13	487	−23	−78	394	−73	*E.dispar*
Sample 14	−75	−69	−81	−78	−71	Negative
Sample 15	530	−11	−65	478	−63	*E.dispar*
Average blank^***^	111	101	116	117	115	

^*^CTL= positive control from cloned-derived PCR biotinylated products.

^**^Real time PCR specific for *E. histolytica* and *E. dispar.*

^***^ From triplicate.

## Discussion

Microscopic examination remains the gold standard method for diagnosing intestinal *Entamoeba* infection despite the fact that it cannot differentiate between *E. histolytica, E. moshkovskii* and *E. dispar*. Moreover, *E. polecki*, *E. coli*, and *E. hartmanii* can be differentiated morphologically from *E. histolytica*, but some of their diagnostic morphologic features overlap depending on the quality of the smears, creating issues for the differential diagnostic identification. Microscopy should actually still be considered as the screening method for the detection of *E. histolytica/E. dispar* complex/genus as well as the other *Entamoeba* found in human stools. However, at this time, *E. histolytica* infections can be easily confirmed with the use of molecular approaches. During the last decade, several PCR-based methods and antigen tests have been developed for the detection of *E. dispar* and *E. histolytica.* However, most of these platforms have certain limitations when considering its use in a multiplex format. Multiplexed detection of *Entamoeba* spp. could strengthen the diagnosis of amebiasis. Recently a multiplex PCR-bead protocol provided a sensitive diagnostic screen for a large panel of intestinal parasites, but included only one species of *Entamoeba*, *E. histolytica*[40].

The main objective of this study was to create a proof of concept for simultaneous DNA-based detection of pathogenic and non-pathogenic amebas. In order to accomplish this goal we developed a Luminex assay for detection and differentiation of *E.* histolytica, *E. dispar*, *E. hartmanii*, *E. coli*, *E. moshkovskii*. In the future such assays can be validated for use in clinical diagnosis.

In our study the PCR products were produced with biotinylated primers and mixed with microspheres that had been coupled to probes designed on 18SrRNA sequences that could differentiate *Entamoeba* species. PCR products, following hybridization with the beads were incubated with streptavidin–*R*-phycoerythrin (SAPE), which binds to the biotin on the PCR amplicons. If the product was present in the sample, each probe-microsphere set would capture the specific PCR product. After that, each microsphere was analyzed in a Luminex instrument capable of identifying specific color signature of each microsphere as well as detecting the SAPE bound to the hybridized PCR products. The primer pair used amplified products that ranged from 622 to 667 bp, according to the *Entamoeba* species. However, the hybridization signals obtained from these amplicons with their respective probes showed low hybridization signals. The conformation of the amplified DNA or the presence of secondary structures in the region of hybridization probes could be the explanation for such results. Previous studies showed that the length of the amplicon could influence the hybridization profiles, especially when showing complex structures prone to the creation of multiple hairpin loops and stem structures [33]. However, Diaz and Fell [44] found a lower hybridization signal with the shortest amplicon target and a higher hybridization signal with amplicon targets of 600 bp and higher. On the other hand, Etienne *et al*. [45] observed that optimal hybridization took place when using amplicons of 250-bp. Other studies used amplified products between 100 and 400 bp to minimize the potential steric hindrance deterrent effect, which affects the efficiency of hybridization [35]. The authors showed that the antisense primer had to be re-designed in order to make smaller amplicons and minimize the chances of secondary structure. JVF/EntaRev390 and JVF/Enta417 primers produced amplified products ranging from 382 to 429 nucleotides. The reverse primer Enta Rev390 has a mismatch of C/T on *E. coli* sequence near the 5′ end. However, the efficiency of amplification was not affected. Some factors affect the specificity and sensitivity of multiplex hybridization such as temperature, time and amount of target DNA. We analyzed 5 μl of biotinylated amplicon diluted with 12 μl of TE buffer (pH 8) and 17 μl of biotinylated amplicon diluted 1:100 in TE in order to avoid concentration beyond the linear range, at or near the initial saturation level without sacrificing discriminations. Optimum hybridization occurred at 46°C for 1 h. PCR based suspension array parameters were determined using cloned DNA fragments amplified from *Entamoeba* spp*.* An additional set of *Entamoeba* positive clinical isolates were tested. The results shown are strengthened by the fact that aliquots of the same sample were used by other molecular tests. In this study, we used stool samples positive by real- time PCR, PCR followed by DNA sequencing analysis and microscopy. There was 100% agreement between the results of these tests and the identification derived by the comparative DNA sequencing analysis, microscopy and real-time PCR combined, thus yielding an assay specificity of 100%. In addition, the PCR-based suspension array assay was able to detect *E. hartmanni* in stool samples that had more than one *Entamoeba* species, but which had not been detected by microscopic analysis and DNA sequencing analysis. Further microscopic evaluation using more refined measurement of the cysts finally revealed the presence of *E. hartmanii*. These data suggest that the multiplex Luminex array was able to provide a very robust discriminatory power in detecting and differentiating *Entamoeba* species, even in mixed infection samples. Concomitant infections with two or more different *Entamoeba* are not uncommon, and their identification may be important for determining the most appropriate therapy and epidemiological data. Moreover, mixed infection requires cloning followed by DNA sequencing, which is currently labor-intensive, time-consuming, and costly. To date, the real-time PCR techniques developed cannot be used to differentiate all *Entamoeba* species found in human stools. There are several merits of the multiplex system compared to the use of microscopy, real-time PCR and DNA sequencing analysis. This system reduces time, and labor required for *Entamoeba* detection. The strength of the assay is that it could also be used to detect the presence of multiple *Entamoeba* species in the same reaction vessel.

## Conclusion

In conclusion, the assay described here represents a proof of concept to supplement traditional microscopy in a routine diagnostic setting. The assay should be a suitable technique for multiplex identification and differentiation of E*. histolytica*, *E. dispar*, *E hartmanni*, *E. coli* and *E. moshkovskii* in fecal samples and can refine the diagnosis of amoebiasis as well as being an important tool in studies designed to ascertain the distribution of different species of *Entamoeba* in human stools.

## Competing interests

The authors have declared that no competing interests exist.

## Authors’ contributions

AJS, JMP, HLCS and RHSP planned and designed the protocols. RB supervised all the laboratory work. HLCS and KB carried out the data analysis and interpretation. HLCS, prepared the first draft of the manuscript and all authors revised the manuscript critically. All authors read and approved the final version of the manuscript.

## Note

The findings and conclusions in this manuscript are those of the authors and do not necessarily represent the views of the Centers for Disease Control and Prevention. The use of trade names is for identification only and does not imply endorsement by the Public Health Service or by the U. S. Dept. of Health and Human Services.
